# Stop codons in bacteria are not selectively equivalent

**DOI:** 10.1186/1745-6150-7-30

**Published:** 2012-09-13

**Authors:** Inna S Povolotskaya, Fyodor A Kondrashov, Alice Ledda, Peter K Vlasov

**Affiliations:** 1 Bioinformatics and Genomics Programme, Centre for Genomic Regulation (CRG) and UPF, 88 Dr. Aiguader, Barcelona 08003, Spain; 2 ICREA, Institució Catalana de Recerca i Estudis Avançats, Lluis Companys, Barcelona, Spain; 3 Inserm U722, Faculté de Médecine Xavier Bichat, 16, rue Henri Huchard, Paris 75018, France

## Abstract

**Background:**

The evolution and genomic stop codon frequencies have not been rigorously studied with the exception of coding of non-canonical amino acids. Here we study the rate of evolution and frequency distribution of stop codons in bacterial genomes.

**Results:**

We show that in bacteria stop codons evolve slower than synonymous sites, suggesting the action of weak negative selection. However, the frequency of stop codons relative to genomic nucleotide content indicated that this selection regime is not straightforward. The frequency of TAA and TGA stop codons is GC-content dependent, with TAA decreasing and TGA increasing with GC-content, while TAG frequency is independent of GC-content. Applying a formal, analytical model to these data we found that the relationship between stop codon frequencies and nucleotide content cannot be explained by mutational biases or selection on nucleotide content. However, with weak nucleotide content-dependent selection on TAG, -0.5 < Nes < 1.5, the model fits all of the data and recapitulates the relationship between TAG and nucleotide content. For biologically plausible rates of mutations we show that, in bacteria, TAG stop codon is universally associated with lower fitness, with TAA being the optimal for G-content < 16% while for G-content > 16% TGA has a higher fitness than TAG.

**Conclusions:**

Our data indicate that TAG codon is universally suboptimal in the bacterial lineage, such that TAA is likely to be the preferred stop codon for low GC content while the TGA is the preferred stop codon for high GC content. The optimization of stop codon usage may therefore be useful in genome engineering or gene expression optimization applications.

**Reviewers:**

This article was reviewed by Michail Gelfand, Arcady Mushegian and Shamil Sunyaev. For the full reviews, please go to the Reviewers’ Comments section.

## Background

Translation termination is a crucial step in protein synthesis that, in most organisms, is triggered by three stop codons; TAA, TGA and TAG. These three stop codons are thought to be functionally equivalent in the broad sense of effective translation termination. Additional functions, such as coding for extra amino acids, effects only a tiny fraction of all codons [1], and these stop codons can be interchanged [2,3] or even lost [4-8] without obvious functional consequences. Indeed, one of the motivations in a recent experimental study of genome-wide codon replacement in selecting to substitute all TAG stop codons in *Escherichia coli,* rather than making synonymous substitutions, was the rationale that synonymous “codon utilization bias has been shown to affect translation efficiency” [3] suggesting that in the author’s opinion stop codon substitution may have fewer functional consequences than synonymous substitution. Thus, at present there is broad consensus that three stop codons are functionally equivalent and interchanging stop codons is not expected to have functional or selective consequences. In that case substitutions between different stop codons should be neutral, such that the rate of evolution between stop codons should be broadly equivalent to the synonymous rate of evolution and the stop codon frequency should be governed by similar selective and mutational forces that govern nucleotide usage in synonymous sites.

The hypothesis of selective equivalence of stop codons has not been rigorously tested and, contrary to the general expectation, there are data that suggest that stop codon may not be entirely synonymous. Firstly, translation termination efficiency may be nucleotide context dependent [9-13]. Second, TAG and TGA stop codon frequencies in bacterial genomes with different GC-contents are strikingly different (see Figure 1 in [14]), such that TGA frequency increases with genomic GC-content while TAG is GC-content independent. Here, we study stop codon frequency and evolution in bacterial genomes to gain an understanding of whether or not stop codons are used indiscriminately without any fitness costs. We compare rates of stop codon evolution to the rate of synonymous evolution and apply a simple population genetics model formulated by Bulmer [15] to stop codon frequency and nucleotide content in bacterial genomes.

**Figure 1:**
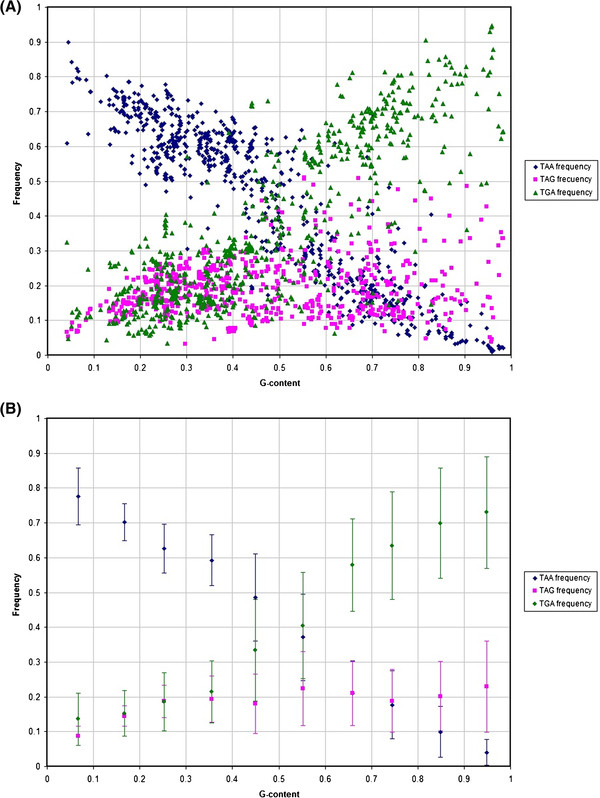
**The relationship between stop codon frequencies in 736 bacterial genomes and G content in GA-type twofold synonymous codons.** The pattern is shown for all genomes (**A**) and as an average measure for bins of 10% of G-content with SD (**B**).

## Results

### Stop codon evolution and frequency

There are two predictions of the synonymous usage of stop codons: stop codon evolution should occur at a rate equivalent to that of synonymous evolution and stop codon frequency should mirror that of synonymous codons, such that AT-rich genomes should show a higher frequency of TAA. The three stop codons are interchangeable through one, or two, transitions of G - > A or A - > G. Thus, when comparing the rate of evolution of the stop codons it is best to use the same transition G < − > A, which occurs between some two-fold synonymous sites: glutamine, glutamic acid and lysine. Similarly, when comparing stop codon frequency it is more appropriate to use G-content at such two-fold sites than genome-wide or four-fold synonymous GC-content.

First, we compared the rate of stop codon evolution (*K*_*stop*_) to synonymous evolution in 11 pairs of bacterial genomes. We found that stop codon evolution, which involves only the G < − > A transitions, is ~1.7 times slower than the rate of synonymous changes in G < − > A two-fold sites, *K*_*GA*_ (*K*_*stop*_/*K*_*GA*_ = 0.58 ± 0.19, SD). However, the difference is not large, such that *K*_*stop*_ is closer to *K*_*GA*_ than *K*_*N*_ is to *K*_*S*_ (*K*_*n*_*/K*_*s*_ = 0.09 ± 0.04, SD) indicating that evolution of stop codons is affected by the action of weak selection or mutational biases. While the observation of *K*_*stop*_ < *K*_*AG*_ is indicative of negative selection acting on substitutions between stop codons, it is by itself not conclusive. It is likely that some form of negative selection is acting on synonymous sites, which in some circumstances increases the rate of evolution [15], thus, *K*_*stop*_ < *K*_*AG*_ may be a consequence of negative selection on synonymous sites [16,17] and additional data are necessary to corroborate the possibility of selection acting on stop codons.

Second, we considered the dependence of the stop codon frequency on guanine content in G/A two-fold degenerate sites of 736 bacterial genomes (Figure 1), following the results of Wong and colleagues (Figure 1 from 14). The lack of a clear correlation between TAG usage and frequency of guanine is particularly striking in comparison to the expected behavior of TGA stop codon. Moreover, it is apparent that TAG stop codon is rarely very frequent in the genomes with the average expected frequency of around 20%, although this frequency is slightly lower in very A-rich genomes (Figure 1B).

**Figure 2:**
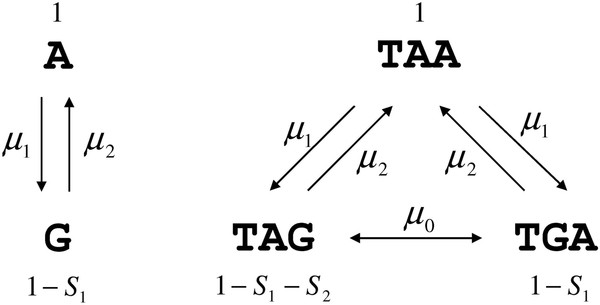
The model, with rates of mutation and selection coefficients on nucleotide content and stop codons.

The independence of TAG on guanine frequency at first glance has a simple explanation, that TAA and TGA stop codons are functionally equivalent while the TAG stop codon performs a different function and almost never evolves into the other two codons. However, this simple explanation for these data is readily refuted by the observation that the rate of TAG stop codon evolution is non-zero and is comparable with the rate of evolution of the other two codons (0.50 ± 0.42, 0.86 ± 0.37, 0.43 ± 0.13 for TAA, TGA and TAG, respectively, with SD), the experimental evidence that TAG can be easily changed without profound consequences [3] and the observation that TAG frequency is the same for all functional categories (Additional file 1: Figure S1). Thus, the lack of a response of TAG to guanine frequency cannot be explained by strong evolutionary conservation of the TAG stop codon in specific genes. Similarly, this effect does not appear to be caused by different propensities of stop codons in overlapping genes (Additional file 2: Figure S2). These data are suggestive of a nontrivial system, such that despite the apparent lack of change of TAG frequency with guanine frequency the rate of TAG codon evolution is not close to zero.

### Model of stop codon evolution

To understand the possible causes of the apparent paradox that in bacteria all three stop codons show substantial rates of evolution while the frequency of the TAG stop codons remains at ~20% independently of the nucleotide content we developed a simple formal model of stop codon and guanine genomic frequency. We applied a model developed by Bulmer [15] for synonymous codon usage and solved it explicitly for the genomic frequency of stop codons, with rates of mutation between them and selection for each stop codon as parameters in the model. This theoretical framework assumes that substitutions are rare and two substitutions rarely segregate at the same time, which fits well to stop codon evolution given the relative rarity of stop codons in bacterial genomes. In this model we use guanine frequency in two fold synonymous sites instead of GC-content of the genome as an independent variable. Two rates of mutation G- > A and A- > G are an explicit part of the model of stop codon evolution (Figure 2) and exactly the same mutations are found in G < − > A two-fold synonymous sites, making it possible to model G-content while using the same mutation rates.

**Figure 3:**
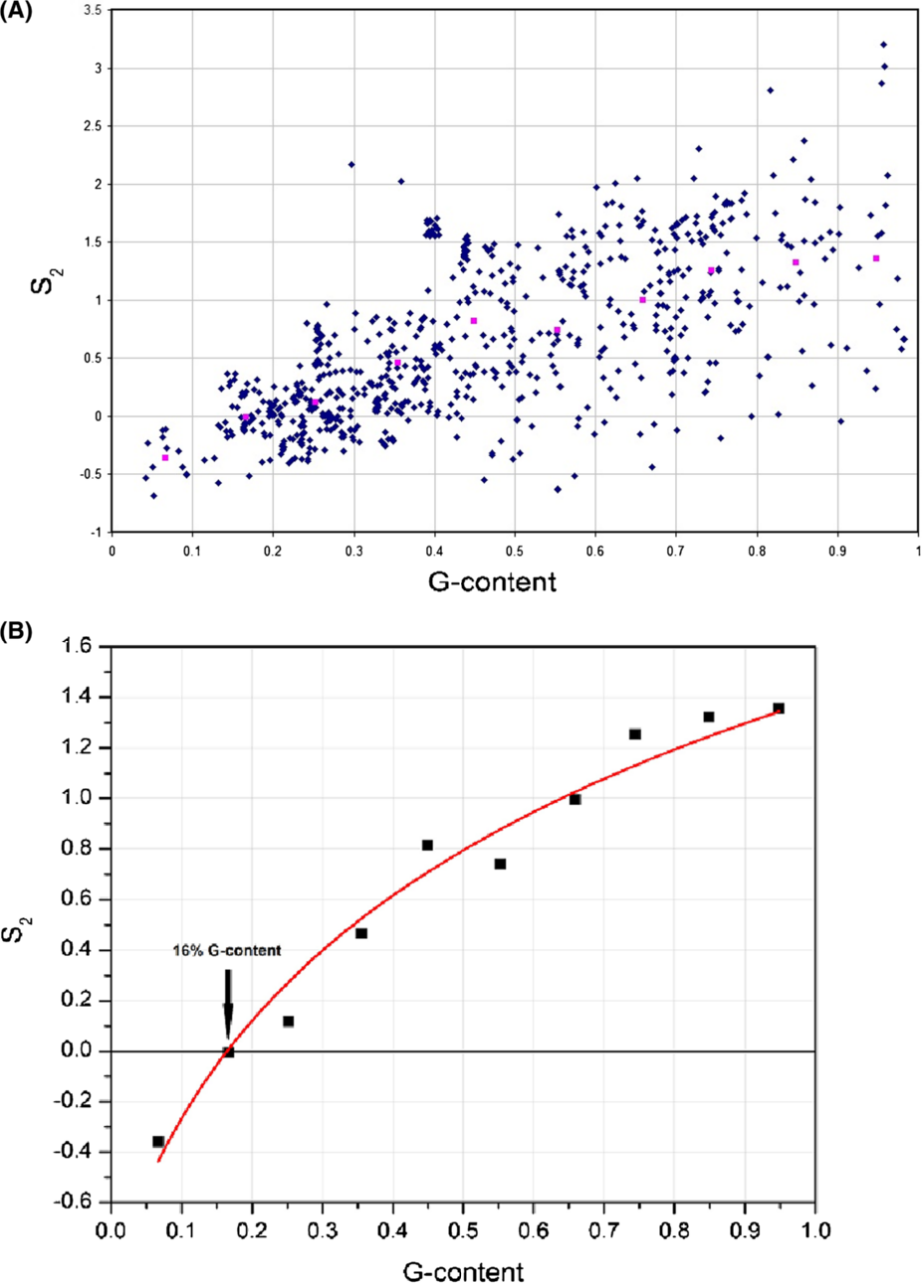
**The estimated selection coefficients on TAG, S2, for individual genome measurements (blue) and the average for bins of 10% in magenta (A).** The average of the estimated values of S2 (black points) and the red line approximating the average estimated values as S2 ~ ln(3.6fG + 0.4) (**B**).

Consider stochastic mutation-selection-drift equilibrium at a locus with three alleles: TAA, TAG and TGA. Assuming for any mutational rate *μ*, *μN*_*e*_ ≪ 1, where *N*_*e*_ is the effective population size, a specific certain allele is fixed most of the time and the frequency of this allele *f* is the fraction of time when it is fixed. The flux of switches to an allele with small selective advantage (*s*) is $\phi =\frac{\mu S}{1-e^{-S}}f$ (eq. 7 from [15]), where $S=2N_{e}s$. Equilibrium allele frequencies can be obtained by solving the following system of linear equations:

(1) $$\left\{ \begin{array}{l} 0=-f_{\text{TAA}}\times (\phi_{TAA>TAG}+\phi_{TAA>TGA})\\ \;+f_{\text{TAG}}\times \phi_{TAG>TAA}+f_{\text{TAA}}\times \phi_{TGA>TAA}\\ 0=f_{\text{TAA}}\times \phi_{TAA>TAG}-f_{\text{TAG}}\\ \;\times (\phi_{TAG>TAA}+\phi_{TAG>TGA})+f_{\text{TAA}}\times \phi_{TGA>TAG}\\ 0=f_{\text{TAA}}\times \phi_{TAA>TGA}+f_{\text{TAG}}\times \phi_{TAG>TGA}-f_{\text{TAA}}\\ \;\times (\phi_{TGA>TAA}+\phi_{TGA>TAG}) \end{array}\right.$$

We consider finesses of every allele to be different, with the selection $S_{1}$ shaping G-content of the genome and selection $S_{2}$ acting on TAG (Figure 2). We assume both selective forces *s*_*1*_ and *s*_*2*_ to be small (*~1/N*_*e*_) and thus the term *s*_1_*s_*2*_ in the expression for the fitness of TAG *(1-s*_*1*_)*(1-s_*2*_*)* is negligible. Another feature of this model is that the rate of mutation A < − > G in the stop codons is identical to the rate of mutation A < − > G in two fold synonymous sites (Figure 2). Overall, there are no reasons why these assumptions are not expected to hold in bacterial genomes so that our model should provide a reasonable approximation of frequencies and selection, if any, of stop codons.

Within the framework of this model we can infer the fluxes between stop codons as:

$$\begin{array}{l} \phi_{TAA>TAG}=\frac{\mu_{1}\left(S_{1}+S_{2}\right)}{\text{exp}\left(S_{1}+S_{2}\right)-1},\\ \phi_{TAG>TAA}=\frac{\mu_{2}\left(S_{1}+S_{2}\right)\text{exp}\left(S_{1}+S_{2}\right)}{\text{exp}\left(S_{1}+S_{2}\right)-1}\\ \phi_{TAA>TGA}=\frac{\mu_{1}S_{1}}{\text{exp}\left(S_{1}\right)-1},\\ \phi_{TGA>TAA}=\frac{\mu_{2}S_{1}\text{exp}\left(S_{1}\right)}{\text{exp}\left(S_{1}\right)-1}\\ \phi_{TAG>TGA}=\frac{\mu_{0}S_{2}}{\text{exp}\left(S_{2}\right)-1},\\ \phi_{TGA>TAG}=\frac{\mu_{0}S_{2}\text{exp}\left(S_{2}\right)}{\text{exp}\left(S_{2}\right)-1} \end{array}$$

and system (1) can be solved analytically:

$$\begin{array}{l} f_{\text{TAA}}=\frac{\frac{\mu_{2}}{\mu_{1}}\text{exp}\left(S_{1}\right)\text{exp}\left(S_{2}\right)}{1+\frac{\mu_{2}}{\mu_{1}}\text{exp}\left(S_{1}\right)\text{exp}\left(S_{2}\right)+\text{exp}\left(S_{2}\right)};\\ f_{\text{TAG}}=\frac{1}{1+\frac{\mu_{2}}{\mu_{1}}\text{exp}\left(S_{1}\right)\text{exp}\left(S_{2}\right)+\text{exp}\left(S_{2}\right)};\\ f_{\text{TAA}}=\frac{\text{exp}\left(S_{2}\right)}{1+\frac{\mu_{2}}{\mu_{1}}\text{exp}\left(S_{1}\right)\text{exp}\left(S_{2}\right)+\text{exp}\left(S_{2}\right)}\text{;} \end{array}$$

Since S_1_ is the selection on G content and mutational rates A < − > G in stop codons are the same as the mutational rates A < − > G in two fold synonymous sites, the equilibrium frequency of G in two fold synonymous sites is the solution of the following system:

(2) $$\left\{ \begin{array}{l} 0=-f_{A}\times \phi_{A>G}+f_{G}\times \phi_{G>A}\\ 0=f_{A}\times \phi_{A>G}-f_{G}\times \phi_{G>A} \end{array}\right.$$

where $\phi_{A>G}=\frac{\mu_{1}S_{1}}{\text{exp}\left(S_{1}\right)-1}\;,\;\phi_{G>A}=\frac{\mu_{2}S_{1}\text{exp}\left(S_{1}\right)}{\text{exp}\left(S_{1}\right)-1}\text{;}$

(3) $$f_{G}=\frac{1}{1+\frac{\mu_{2}}{\mu_{1}}\text{exp}\left(S_{1}\right)}$$

The expressions for the frequencies of stop codons could thus be rewritten as:

(4) $$\begin{array}{l} f_{\text{TAA}}=\frac{\frac{\mu_{2}}{\mu_{1}}\text{exp}\left(S_{1}\right)\text{exp}\left(S_{2}\right)}{1+\frac{\mu_{2}}{\mu_{1}}\text{exp}\left(S_{1}\right)\text{exp}\left(S_{2}\right)+\text{exp}\left(S_{2}\right)}\\ \;=\frac{\left(\frac{1-f_{G}}{f_{G}}\right)\text{exp}\left(S_{2}\right)}{1+\left(\frac{1}{f_{G}}\right)\text{exp}\left(S_{2}\right)}f_{\text{TAG}}\\ \;=\frac{1}{1+\frac{\mu_{2}}{\mu_{1}}\text{exp}\left(S_{1}\right)\text{exp}\left(S_{2}\right)+\text{exp}\left(S_{2}\right)}\\ \;=\frac{1}{1+\left(\frac{1}{f_{G}}\right)\text{exp}\left(S_{2}\right)}f_{\text{TAA}}\\ \;=\frac{\text{exp}\left(S_{2}\right)}{1+\frac{\mu_{2}}{\mu_{1}}\text{exp}\left(S_{1}\right)\text{exp}\left(S_{2}\right)+\text{exp}\left(S_{2}\right)}\\ \;=\frac{\text{exp}\left(S_{2}\right)}{1+\left(\frac{1}{f_{G}}\right)\text{exp}\left(S_{2}\right)} \end{array}$$

Next, we investigate the behavior of this model by starting with its simplest possible modification. Such modification is done by setting parameters to the value of zero, which allows us to trace the impact of each parameter. First, we investigated the model without any selection where all stop codon confer equal fitness and G frequency is determined solely by mutational pressure (S_1_ = S_2_ = 0). In this case

$$\begin{array}{l} f_{G}=\frac{1}{1+\frac{\mu_{2}}{\mu_{1}}},f_{\text{TAA}}=\frac{1-f_{G}}{1+f_{G}},\\ f_{\text{TAG}}=\frac{f_{G}}{1+f_{G}},f_{\text{TAA}}=f_{\text{TAG}}=\frac{f_{G}}{1+f_{G}}\text{;} \end{array}$$

Thus, if there is no selectional pressure the expected frequencies of TAG and TGA are equal and, therefore, a model without any selection cannot fit our data (Figure 1).

Next, we investigated the impact of selection *S*_*1*_ which shapes G-content. Three parameters*, μ*_*1*_, *μ*_*2*_ and *S*_*1*_ act as one effective parameter in the expressions of stop codon frequencies: $\frac{\mu_{2}}{\mu_{1}}\text{exp}\left(S_{1}\right)=\frac{1-f_{G}}{f_{G}}$ from (3). Thus, selection on G-content, *S*_*1*_, affects only G-content itself and does not change the form of the relationship between G frequency and stop codon usage as is evident from expressions (4).

In order to estimate the strength of selection acting on TAG we solve the system of equations (4) for the selection coefficient *S*_*2*_ :

Now we can estimate the value of *S*_*2*_ based on the observed frequencies of TAG, solving the equation (5) for the selection coefficient:

(5) $$S_{2}=\text{ln}\left(\frac{f_{G}\left(1-f_{\text{TAG}}\right)}{f_{\text{TAG}}}\right)$$

Both G-content *f*_*G*_ and frequency of TAG *f*_*TAG*_ are measured directly and for every genome we calculate the predicted value *S*_*2*_ using expression (5) (Figure 3A). *S*_*2*_ has a clear G-content dependence, which can be approximated by $S_{2}\approx \text{ln}\left(3.6f_{G}+0.4\right)$ (5). The predicted value of *S*_*2*_ changes between −0.5 for A-rich and 1.5 for G-rich genomes, respectively (Figure 3B). Using this approximation of *S*_*2*_ we obtain the following expressions for stop codon frequencies:

$$\begin{array}{l} f_{\text{TAA}}=\frac{\left(\frac{1-f_{G}}{f_{G}}\right)\left(3.6f_{G}+0.4\right)}{1+\left(\frac{1}{f_{G}}\right)\left(3.6f_{G}+0.4\right)};\\ f_{\text{TAG}}=\frac{1}{1+\left(\frac{1}{f_{G}}\right)\left(3.6f_{G}+0.4\right)};\\ f_{\text{TAA}}=\frac{\left(3.6f_{G}+0.4\right)}{1+\left(\frac{1}{f_{G}}\right)\left(3.6f_{G}+0.4\right)}\text{;} \end{array}$$

**Figure 4:**
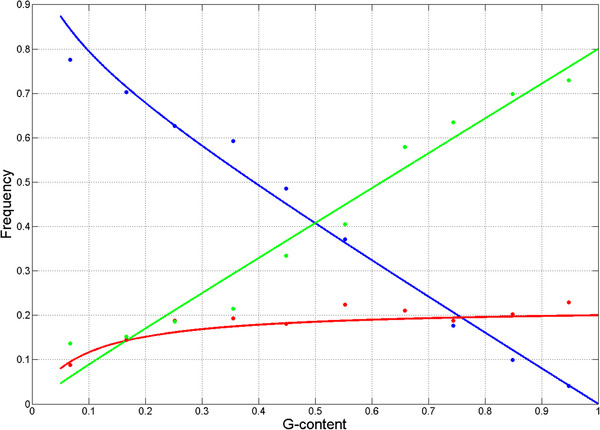
**Expected stop codon frequencies based on expressions (4) with the approximation**$S_{2}\approx \ln(3.6f_{G}+0.4\text{bf})$**.** Points represent average observed stop codon frequencies for TAA (blue), TGA (green) and TAG (red) across binds of 10% G-content while the approximations are shown with the lines.

These expressions recapitulate the relationship between the frequency of TAG and nucleotide content (Figure 4). Thus, the observed frequencies of stop codons in bacterial genomes can be explained only if stop codon are not selectively equivalent, with weak negative selection acting on TAG codon for G-content >16% and weakly positive selection for these two codon when G-content <16%.

**Figure 5:**
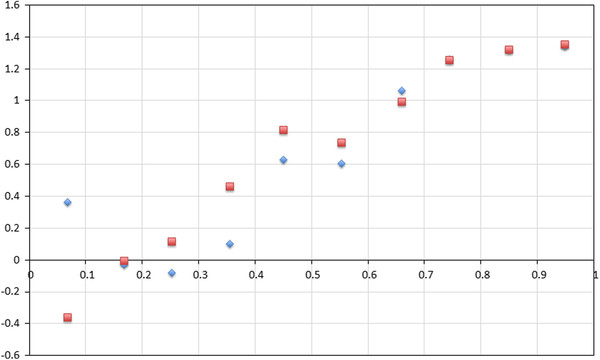
**The predicted values of *S***_***2***_**based on the *f***_***TAG***_**and *f***_***G***_**,**$S_{2}=\ln\left(\frac{f_{G}\left(1-f_{\text{TAG}}\right)}{f_{\text{TAG}}}\right)$**(red) and based on *f***_***TAG***_**and *f***_***TGA***_**, *S***_***2***_ ***= ln (f***_***TGA***_***/f***_***TAG***_***) *(blue)**.

In our model we assumed the same selection regime shaping G-content in coding regions and in stop codons. However, the selective disadvantage of *S*_*2*_ holds when this assumption is removed from the model. Specifically, as could be seen from (4), *exp(S*_*2*_*) = f*_*TGA*_*/f*_*TAG*_, such that we can solve for S2 only based on the comparison of TAG and TGA frequencies. The predicted values of *S*_*2*_ based on this formula are similar to the predicted values based on formula (5) (Figure 5) except for the genomes of low G content (<16%). This, the predicted selection coefficient *S*_*2*_ is positive for nearly all ranges of G content, indicating that the TGA stop codon provides a selective advantage in comparison with the TAG stop codon.

## Discussion

**Figure 6:**
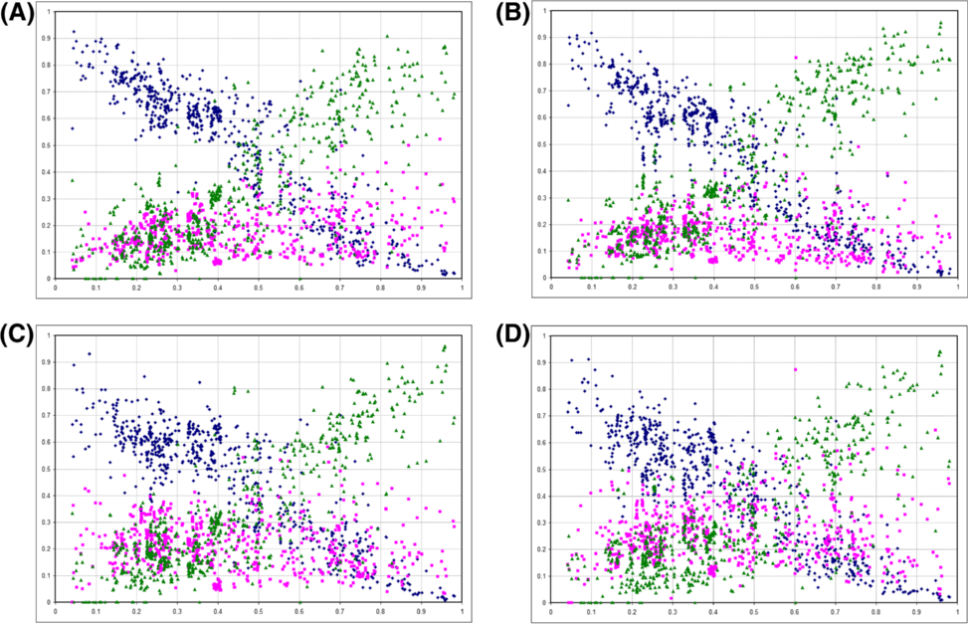
**The relationship between stop codon frequencies in 736 bacterial genomes and G content in GA-type twofold synonymous codons.** The pattern is shown for all codons with A **(A)**, T **(B)**, G **(C)** and C **(D)** nucleotides in the position immediately posterior to the stop codon.

The relationship between stop codon frequency and G-content, or GC-content as reported previously [14], is one of the most striking and unambiguous patterns in bacterial genome composition (Figure 1). Here, we have developed a simple model that captures all of the major observations of stop codon distribution across bacterial genomes. However, as with many theoretical treatments our model necessarily makes several simplifying assumptions. First, we ignore the effects of neighboring nucleotides while, in principle, co-evolution of the nucleotide immediately after the stop codon may be effecting TAG frequency more than that of TGA and TAA frequencies through either contextually-dependent mutational effects [18,19] or by affecting the efficiency of stop-codon recognition [9-12]. However, the lack of a relationship of TAG frequency and G content does not depend on the nucleotide immediately after the stop codon (Figure 6) indicating that the nucleotide context is not an important factor in explaining the pattern of stop codon usage (Figure 1). Second, the same stop codons can be subject to different selection pressures in different genes due to difference in the levels of expression [20] or other factors. To alleviate the fears that a more general model, one that takes into account the distribution of selection coefficients in a genome, would substantially alter our conclusions we have analyzed the effect of assuming a distribution of selection coefficients. We have shown that if a given frequency of the TAG stop codon is explained by a distribution of selection coefficients then the expected value of the average selection of such a distribution would have to be greater or equal to a selection coefficient that is uniform across all TAG codons and leads to the same TAG frequency in the genome (see Methods). In other words, differences in the strength of selection across different TAG codons make our argument stronger that, on average, the TAG stop codon is unpreferred.

Using a population genetics model modified to describe stop codon and guanine frequencies we demonstrated that stop codon usage can be explained when selection is acting specifically on TAG. The predicted selection regime on TAG, *S*_*2*_, has three properties: it is relatively weak, with *N*_*e*_*s* between −0.5 and 1.5, nucleotide content dependent and is positive when G-content <16% and negative when G-content is >16%. The predicted selection strength is weak, on the order of *1/N*_*e*_, which is not strong enough to severely restrict the rate of evolution of stop codons. Indeed, such weak selection on individual alleles can be overpowered by genetic drift, which may result in the large variability of stop codon frequencies in our data (Figure 1B). Alternatively, the observed variability of stop codon frequencies relative to the average expectation (compare Figure 1A and 1B) may be due to slight changes in selection pressure on TAG and the rates of A < − > G mutation between different species (Figure 3A).

The G-content dependence of the selection follows from the roughly constant TAG frequency relative to G-content. Yet at this point, there are no known molecular mechanisms that may explain why TAG stop codon has different selective consequences depending on nucleotide content. One possibility, however, is the dependence of translation termination efficiency on the nucleotide context in the vicinity of the TAG stop codon. Bacteria generally code for two release factors (RF), RF1 that recognizes TAA and TAG stop codons and RF2 that recognizes TAA and TGA [21]. Thus, the prediction of the context-dependence hypothesis is that the efficiency of RF1 is GC-context dependent while RF2 functions independent of nucleotide context. Empirical evidence may be necessary to confirm or refute this hypothesis, however, given the relatively weak nature of the selection the differences in translation termination efficiency may be too small to be easily detected in the laboratory. The possibility of the molecular mechanism involving elongation termination factors, however, is left necessarily uncertain by conflicting data from other species. Eukaryota, that have only one release factor for all three stop codons [22,23], and chloroplast genomes that have retained orthologs of both release factors [24], show a clear increase of TAG frequency with higher GC-content (Additional file 3: Figures S3 and Additional file 4: Figure S4). Clearly, further experimental work is likely necessary to elucidate the molecular mechanisms behind selection on TAG stop codon in bacteria.

Within the framework of our model it is possible to compare the fitness impacts of different stop codons depending on genomic nucleotide content. Regardless of selection on G-content itself (*S*_*1*_) the difference in fitness between TAG and TGA stop codon is defined by *S*_*2*_ (Figure 2). Thus, regardless of the value of *S*_*1*_ our data signify that TAG stop codon is always less fit that the TGA stop codon for G-content >16%. Comparing the relative fitness of TAG and TAA, however, involves both S_1_ and S_2_, with their sum being the difference in relative fitness of these two stop codons (Figure 2). Within the model, G-content depends on relative rates of mutation A < − > G and *S*_*1*_, and we cannot disentangle the contribution of mutation ($\mu_{1}$and $\mu_{2}$) versus selection (*S*_*1*_) so we cannot analytically estimate the value of *S*_*1*_. However, we can define the range of values of these parameters for specific G-content.

**Figure 7:**
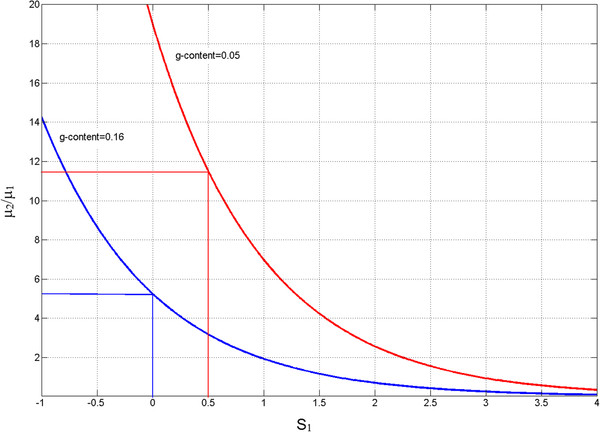
**The relationship between the ratio μ**_**2**_**/μ**_**1**_**, and S**_**1**_**for G-content of 5% (red) and 16% (blue) with the values of the parameters at which there is no selective difference between TAG and TAA (S**_**1**_**-S**_**2**_ **= 0) are indicated by straight lines.**

To identify the plausible range of *S*_*1*_ values in bacterial genomes we investigate the values of $\mu_{2}/\mu_{1}$ and *S*_*1*_ for G-content of 16%. We find that in order for the G < − > A twofold sites to maintain 16% G-content either the rate of G > A mutation must be at least five times larger than the rate of A > G mutations or, *S*_*1*_ must be positive (Figure 7). Thus, for genomes with ~16% G-content TAG can be the stop codon with the highest fitness only if G > A rate of mutation is five times higher than the rate of A > G mutation. For G-content of 5% TAA would confer higher fitness than TAG if S_1_ < 0.5. When S_1_ = 0 the ratio of G < − > A mutations ($\mu_{2}/\mu_{1}$) must be ~19, while a smaller ratio implies a positive S_1_ (Figure 7). When S_1_ = 0.5 the ratio $\mu_{2}/\mu_{1}$ must be at least 11, implying that if $\mu_{2}/\mu_{1}$ < 11 then S_1_ > 0.5 and TAA is more fit than TAG (Figure 7).

Is there any evidence that G > A can be five or ten times faster than A > G mutations? Mutational biases against GC-content that have been measured were shown to be always less than tenfold in favor of AT-content and less than fivefold for 151 out of a total of 154 species considered in two separate studies (25, 26). Similarly, weak selection acting on GC-content has been postulated by several researchers (25,26). Given this evidence it is unlikely that the observed GC-content can be explained solely by G < − > A mutational biases and, therefore, *S*_*1*_ is positive and > > 0.5 for G-content ≈ 5% and >0 for G-content ≈ 16%. Thus, for G-content < 16% the TAG stop codon is expected to be less fit than the TAA stop codon.

## Conclusions

The relative fitness of TAG to TAA and TGA stop codons can thus be described as follows. When G-content is >16% TAG has lower fitness than TGA. As long as *S*_*1*_ *> −S*_*2*_ for G-content <16% then TAG has lower fitness than TAA in bacterial genomes with G-content < 16%. Because *S*_*1*_ *> −S*_*2*_ is expected to hold for G-content < 16% given the mutation parameters observed in nature [25,26] it follows that TAG is a striking example of a global suboptimal codon, such that the substitution of TAG into either TAA or TGA for any bacterial species would lead to an increase of fitness. The use of suboptimal synonymous codons in bacteria is a well-documented phenomena, however, the exact codons that are suboptimal differ substantially between different species (see [27] for review). To our knowledge, the observation that one codon with synonymous function to other codons is always worse in such a large group of organisms, bacteria, is the first example of a global sub-optimality of the genetic table. The sub-optimal organization of the genetic table revealed here provides a striking counterexample to the remarkable optimization of the genetic code with respect to error minimization [28-30].

## Methods

All available complete bacterial genomes were downloaded from NCBI website and 736 of those that utilize the standard genetic code were used for the analysis (See Additional file 5). Plasmid sequences were excluded. All available pairs of closely related genomes from the ATGC database [31], of which there were 11 pairs with 0.03 < *K*_*S*_ < 0.22 were used to measure A < − > G synonymous transition rates (*K*_*AG*_) and rates of stop codon evolution (*K*_*stop*_). Orthologues were constructed using two-directional best BLAST [32] hit approach and aligned using MUSCLE [33]. To obtain *K*_*AG*_ we looked at the number of synonymous differences between three pairs of codons: CAA and CAG, AAA and AAG and GAA and GAG. The expected number of substitutions occurred was estimated using Jukes-Cantor model [34]. The same method was applied to estimate the number of substitutions between stop codons with the only difference that the number of synonymous sites for TAA codon is twice as high as the number of synonymous sites for TAG and TGA codons. In order to obtain rates of TAG codon evolution the substitutions have to be polarized and for that the third organism was added to the 11 pairs of the genomes such that the synonymous distance between sister species 0.02 < *K*_*S*_ < 0.15 and between sister species and outgroup 0.04 < *K*_*S*_ < 0.62. Substitutions were polarized using simple parsimony approach.

To show that a distribution of selection coefficients for the same stop codon across different genes can only increase the differences between average selection coefficients of stop codons we proved the following conjecture. A given frequency of TAG codon in the genome can be explained by an equal strength of selection acting on all TAG codons in the genome ($S_{0}$) or a distribution of selection coefficients across different codons with an expected value of the distribution ($\bar{S}$). For any given observed frequency of the TAG codon in the genome $S_{0}\leq \bar{S}$, such that the average strength of selection in a distribution is larger when different codons are under different selection pressures. We consider the case where selection on each TAG stop codon is a discrete random variable which assumes the value *S*_*i*_ with the probability *p*_*i*_ . In this case we use *S*_*i*_ as discrete values of a distribution of selection coefficients on TAG stop codons in different genes in the same genome, while *S*_*1*_ and *S*_*2*_ were used as fixed values of the selection coefficients for all genes across a single genome. In this case for any selection *S*_*i*_ the expected number of the sites under this selection is *N*_*i*_ *= p*_i_*N_*stop*_*,* the frequency of TAG is $f_{i}^{\text{TAG}}=\frac{1}{1+\frac{\text{exp}\left(S_{i}\right)}{f_{G}}}$ and the number of TAG stop codon is $N_{i}^{\text{TAG}}=f_{i}^{\text{TAG}}*p_{i}*N_{\text{stop}}$. The observed frequency of TAG in the genome is $f_{\text{TAG}}=\frac{\sum_{i}N_{i}^{\text{TAG}}}{N_{\text{stop}}}=\sum_{i}p_{i}*f_{i}^{\text{TAG}}$ and the value of selection *S*_*0*_ acting on TAG sites is estimated from the formula $f_{\text{TAG}}=\sum_{i}p_{i}*f_{i}^{\text{TAG}}=\frac{1}{1+\frac{\text{exp}S_{0}}{f_{G}}}$. Taking into account that the second derivative of *f*, $f^{\prime \prime }=\frac{\frac{\text{exp}S}{f_{G}}\left(\frac{\text{exp}S}{f_{G}}-1\right)}{{\left(1+\frac{\text{exp}S}{f_{G}}\right)}^{3}}\geq 0$, if *S* ≥ 1n*f*_G_, the Jensen’s inequality $\sum_{i}p_{i}*f_{i}^{\text{TAG}}\geq f_{\text{TAG}}\left(\bar{S}\right)$ holds, or $\frac{1}{1+\frac{\text{exp}\bar{S}}{f_{G}}}\leq \frac{1}{1+\frac{\text{exp}S_{0}}{f_{G}}}$ and $S_{0}\leq \bar{S}$. The only condition for this inequality to hold is *S* ≥ 1n*f*_G_, which is a reasonable assumption taking into account the fact that out of 736 genomes analyzed *S*_0_ ≥ 1n*f*_G_ for 734 (Additional file 6: Figure S5).

## Competing interests

The authors declare that they have no competing interests.

## Authors’ contribution

ISP carried out the modeling studies, performed the statistical analysis and wrote the manuscript. AL participated in the statistical analysis and proposed the improvements for the final model. FAK conceived of the study, participated in its design and coordination and wrote the manuscript. PKV conceived of the study, helped with the statistical analysis and helped to draft the manuscript. All authors read and approved the final manuscript.

## Reviewers’ comments

**Reviewer 1: Dr Mikhail Gelfand,Institute for Information Transmission Problems, RAS, Bolshoi Karetny per. 19, Moscow 127994, Russia and Faculty of Bioengineering and Bioinformatics, Moscow State University, Vorobievy Gory 1-73, Moscow 119992, Russia.gelfand@iitp.ru**

The authors present a model explaining the following observation: while the use of the UGA stop codon depends on G-content, the UAG frequency is almost constant in genomes with highly diverse G-content. While I see no problems with the observations and the model, I have some editorial comments and questions.

The authors state several times – starting with the very first sentence of the abstract – that the usage of stop codons has not been rigorously studied. This is not correct. In the 90’s, several papers considered the usage of stop codons and its dependence on the local context, including tandem stops and tetranucleotides involving stop-codons. I think these papers should be mentioned.

**Author response:** Indeed, the term “usage” in this context is not very precise. We acknowledge that there have been studies of stop codon usage in the local context, that is to say that some stop codons have a preferred local context, however, in this manuscript we discuss only the evolution and genomic frequencies of the three different stop codons, which to our knowledge has not been rigorously considered previously. We cite some of the relevant literature and use the word “frequency” which we believe is not as ambiguous as “usage” in this context.

How the 11 studied genome pairs were selected?

**Author response:** We selected all genome triplets with 0.03 < K_S_ < 0.22 that were available in the ATGC database. We now report this in the Methods section.

Is the G/A content the same in the 3rd codon position in all codon pairs? If not, why this is a good parameter?

**Author response**: There are three pairs of two-fold degenerated codon families: AAG/A, GAG/A, CAG/A. G-content at the third position of every pair is indeed highly correlated with overall G-content (see the figure below).

Dependency between G content in the third position of two-fold degenerated codon families and overall G content for AAG/A (blue), GAG/A (red), CAG/A (green).

And in any case, what are the reasons to suspect that the selection regime in the amino-acid-encoding codons is the same as in the stops (the former may depend on concentrations of tRNAs and the codon-anticodon interactions; the latter, on interactions with the release factors). What about the A/G choice in the four-fold codon families?

**Author response:** Indeed, we have created the model based on this assumption because it allowed us to reduce the number of parameters and make the system of equations solvable. However, we can also show that this assumption does not affect our main result that the TAG codon is selectively disadvantageous. Specifically, from system of equations (4) it follows that exp(*S*_*2*_) = *f*_*TGA*_/*f*_*TAG*_. Thus, we can solve for the selective impact of TAG (*S*_*2*_) solely based on the frequencies of TAG and TGA without making the assumption that the selective regime is the same in stop and amino acid codons. Since S2 is positive for almost the entire range of G content it follows that the TAG codon provides a selective disadvantage relative to the TGA codon. Unfortunately, we cannot estimate *S*_*2*_ by comparing the frequencies of TAG and TAA codons because we cannot independently estimate the $\frac{\mu_{2}}{\mu_{1}}\text{exp}(S_{1})$ component of f_TAA_ from (4). We now present the new estimate of *S*_*2*_ in Figure 5 and the main text.

The reasoning in page 6 is not clearly presented, and misprints add to the confusion. How is formula S2 = ln ((fG(1-fTAG))/fTAG) used? Do I understand it correctly that the next formula S2 = ln (3.6fG + 0.4) results from a fit to observations (comparison of genome pairs)? – I think, this should be explained more explicitly.

**Author response:** Yes, this is what we mean, and we rewrote this section to hopefully make this clearer.

By the way, the two formulas for S2, theoretical and observed ones, yield a dependence between fG and fTAG – does it hold?

**Author response:** Yes, there is a slight dependence as can be seen from Figure 1.

*Finally, reference to equation (5) in the preceding paragraph should be about equation (4), and the sentence “S2 has a clear G-content dependence is well approximated…” probably should be “S2 has a clear G-content dependence that is well approximated…” .*

**Author response:** If the referee means this sentence “Thus, selection on G-content,, affects only G-content itself and does not change the form of the relationship between G frequency and stop codon usage as is evident from expressions (4).” then we mean that in the system of equations (4) G-content (f(taa,tga,tag) does not depend on S1. The other typo is corrected.

Polarization of substitutions using parsimony may be dangerous if there is selection towards a specific, preferred nucleotide: in some cases two parallel nonpreferred-to-preferred substitutions may occur, and they will be interpreted as a single preferred-to-nonpreferred substitution, hence skewing the substitution statistics.

**Author response:** This is true, however, these data has been obtained for a number of species with different GC-content and low sequence divergence. Therefore, we believe that it is unlikely that the use of parsimony have produced a systematic error of substantial effect that jeopardizes our conclusions.

**Reviewer 2: Dr. Arcady Mushegian,Stowers Institute for Medical Research, Kansas City, Missouri, United States of America and Department of Microbiology, Kansas University Medical Center, Kansas City, Kansas, United States of America.arm@stowers.org**

The manuscript by Povolotskaya et al. puts forward a simple model of nucleotide substitutions in the stop codons in bacteria, and tests it against the genome-wide data. One of the main conclusions is that TAG may be globally suboptimal, with each of the remaining two codons turning out more fit under different values of GC content.

One biological explanation of these data may be in the phenomenon of overlapping ORFs in bacterial operons. TAG is the only codon that does not accommodate a minimal overlap, whereas TAA can give one kind of stop-start codon overlap (TAATG) and TGA even two kinds (ATGA and TGATG). Perhaps if the authors restricted their sample to the termination codons in the last (or only) genes in operons, they would see much less difference between fitness of those two and TAG?

**Author response:** The idea that the observed pattern of stop codon frequency in bacterial genomes is explained by gene overlap has occurred to us as well. However, we observe the same relationship between G-content and stop codon frequency in overlapping and non-overlapping genes. We now report these data in a new figure that is Additional file 2 Figure S2 in the new version of the manuscript. We have considered only tail-to-tail overlaps due to a much higher certainty of stop codon annotation compared to the uncertainty in the annotation of many start codons.

**Reviewer 3: Dr. Shamil Sunyaev,Dr. Shamil Sunyaev, Division of Genetics, Brigham and Women's Hospital, Harvard Medical School, 77 Ave. Louis Pasteur, Boston MA 02115, USA. ssunyaev@rics.bwh.harvard.edu**

This manuscript presents an analysis of stop codon usage in bacterial species.

The authors report that TAG codon is un-preferred in most bacterial species and that its frequency does not depend on GC content. They suggest presence of weak selection against TAG codon due to unknown mechanism. One potential mechanism may involve dependency of efficiency of one of the release factors on GC content. I find the results of great interest. I only have two minor technical comments.

1) The analysis is based on Bulmer equations, which hold only if evolution is mutation limited. It would be great to briefly discuss applicability of this model to a wide variety of bacterial species.

**Author response:** Bulmer’s model assumes that the fate of a new mutation is decided independently of other mutations, that is to say that generally only one mutation is segregating in the population at the same time. This is certainly true if we consider only mutations in stop codons. In most bacterial genomes there are 2–5 thousand protein coding genes making it rather unlikely that more than one stop codon polymorphism is segregating at the same time.

2) Approximation of selection coefficient against TAG codon as a sum of contributions due to selection against GC content (S1) and selection against this specific codon (S2) ignores the S1*S2 term. It is OK if both selective forces are assumed to be small. It would be great if this assumption would be spelled out.

**Author response:** The referee is absolutely correct, we assume that both of the selective forces are small. We have added an explicit statement to this effect in the text.

## Supplementary Material

Additional file 1 **Figure S1.** The frequency distribution of protein function families, Clusters of Orthologous Groups, relative to the frequency of stop codons. Click here for file

Additional file 2 **Figure S2.** Stop codon frequencies for all genes and for non-overlapping genes in tail-to-tail orientation. Click here for file

Additional file 3 **Figure S3.** Stop codon frequencies in 118 Chloroplast genomes. Click here for file

Additional file 4 **Figure S4.** Stop codon frequencies in nuclear genomes of 62 Eukaryotes. Click here for file

Additional file 5 **Table S1.** Identifiers and summary statistics on stop codon frequencies, fourfold and GA twofold synonymous sites for all bacterial genomes. **Table S2:** Estimates of the rate of evolution in different sites between pairwise comparisons of closely related species. **Table S3:** Estimates of the rate of evolution of stop codons between two closely related species polarized by a closely related outgroup. **Table S4:** Identifiers and summary statistics on stop codon frequencies and fourfold site nucleotide composition in nuclear Eukaryotic genomes. **Table S5:** Identifiers and summary statistics on stop codon frequencies and fourfold site nucleotide composition in Chloroplast genomes. Click here for file

Additional file 6 **Figure S5.** Distribution of selection coefficients associated with TAG frequency in 736 bacterial genomes. The area above the red line represents cases when $S_{0}\geq \text{ln}f_{G}$. Click here for file
