# Supplementary figures and images for: Stop codons in bacteria are not selectively equivalent
Source: Biol Direct. 2012 Sep 13;7:30. doi: 10.1186/1745-6150-7-30 (PMC3549826; doi:10.1186/1745-6150-7-30)

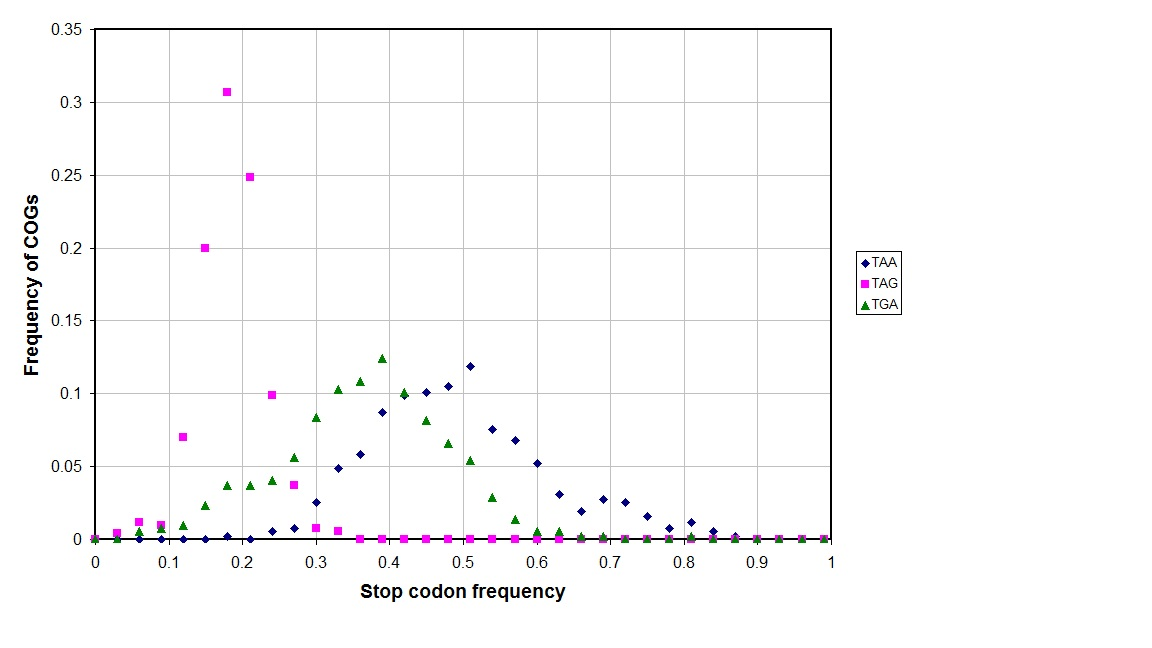

Supplement: Additional file 1 — Figure S1. The frequency distribution of protein function families, Clusters of Orthologous Groups, relative to the frequency of stop codons. [file 1745-6150-7-30-S1.tiff]

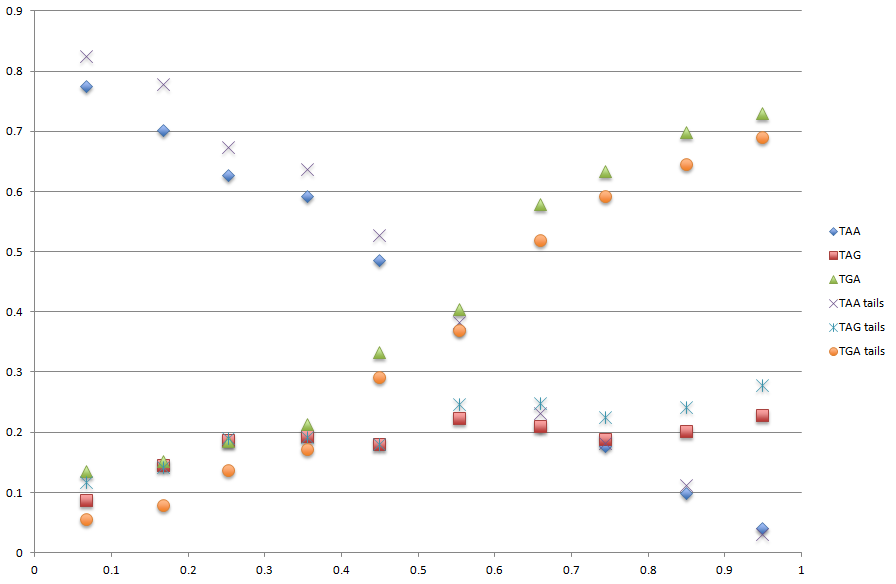

Supplement: Additional file 2 — Figure S2. Stop codon frequencies for all genes and for non-overlapping genes in tail-to-tail orientation. [file 1745-6150-7-30-S2.tiff]

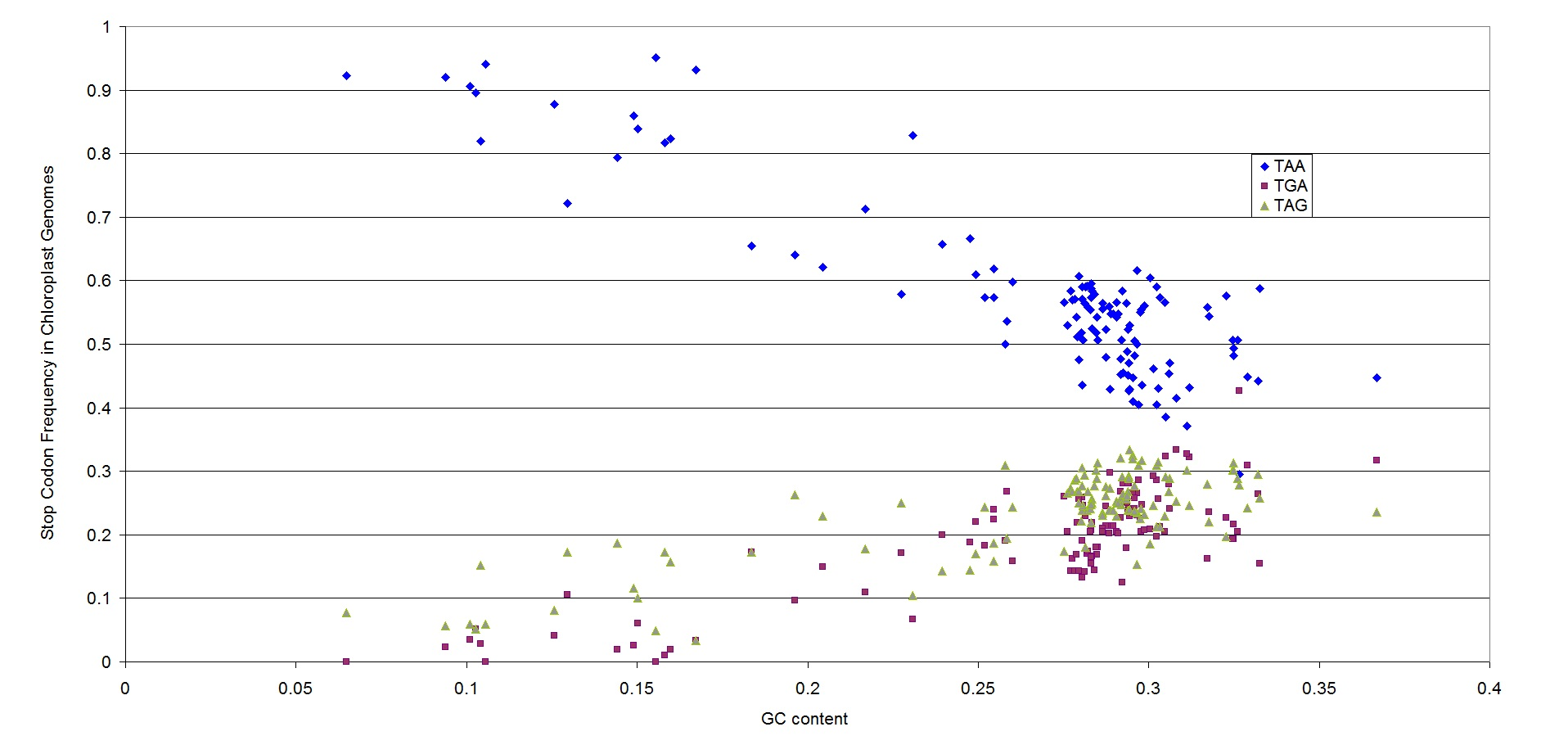

Supplement: Additional file 3 — Figure S3. Stop codon frequencies in 118 Chloroplast genomes. [file 1745-6150-7-30-S3.tiff]

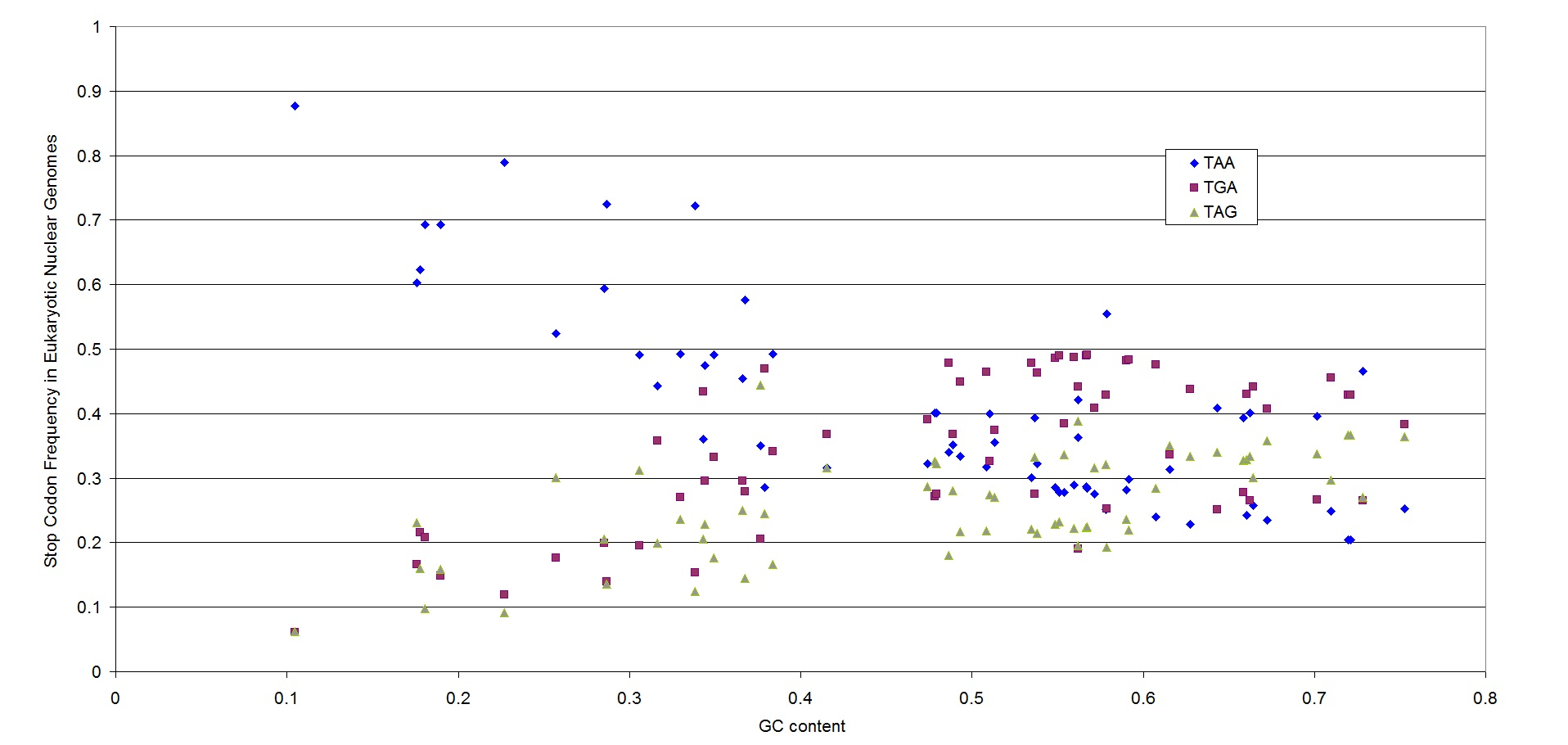

Supplement: Additional file 4 — Figure S4. Stop codon frequencies in nuclear genomes of 62 Eukaryotes. [file 1745-6150-7-30-S4.tiff]

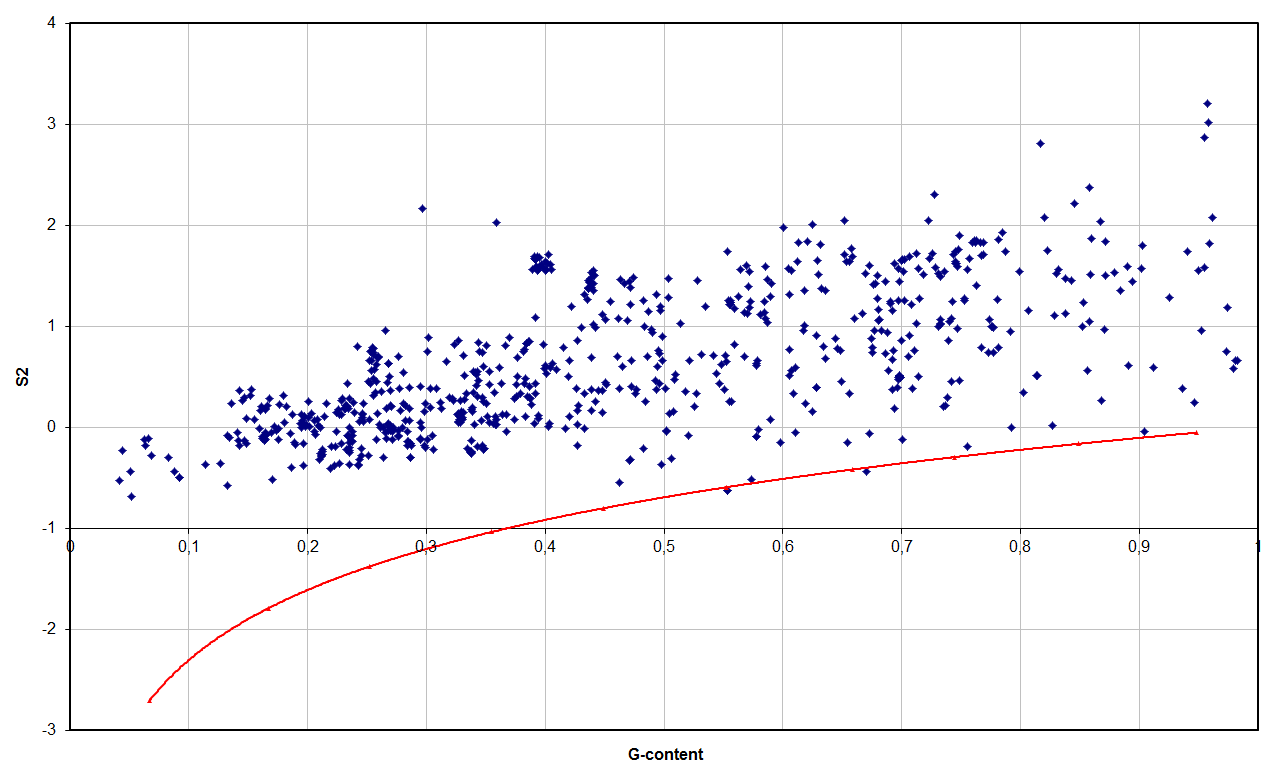

Supplement: Additional file 6 — Figure S5. Distribution of selection coefficients associated with TAG frequency in 736 bacterial genomes. The area above the red line represents cases when S0 ≥lnfG. [file 1745-6150-7-30-S6.tiff]
